# Processing Sentences With Multiple Negations: Grammatical Structures That Are Perceived as Unacceptable

**DOI:** 10.3389/fpsyg.2019.02346

**Published:** 2019-10-22

**Authors:** Iria de-Dios-Flores

**Affiliations:** ^1^English and German Department, Universidade de Santiago de Compostela, Santiago de Compostela, Spain; ^2^Basque Center on Cognition, Brain and Language, Donostia, Spain

**Keywords:** multiple negation, double negation, acceptability, grammatical illusions, interference, negative polarity items, processing complexity

## Abstract

This investigation draws from research on negative polarity item (NPI) illusions in order to explore a new and interesting instance of misalignment observed for grammatical sentences containing two negative markers. Previous research has shown that unlicensed NPIs can be perceived as acceptable when occurring soon after a structurally inaccessible negation (e.g., *ever* in *The bills that no senators voted for have ever become law). Here we examine the opposite configuration: grammatical sentences created by substituting the NPI *ever* with the negative adverb *never* (e.g., *The bills that no senators voted for have never become law*). The processing and acceptability of these sentences were studied using three tasks: a speeded acceptability judgment (Experiment 1), a self-paced reading task (Experiment 2), and an offline acceptability rating (Experiment 3). The results are consistent across measures in showing that the integration of the adverb *never* is disrupted by the linearly preceding but structurally inaccessible negative quantifier *no* in the relative clause. In our view, this pattern of results is in line with Parker and Phillips’ (2016) proposal that NPI illusions arise when the context containing the inaccessible negation has not been fully encoded by the time the NPI *ever* is encountered, making the embedded negative quantifier transparently available as a licensor. In a similar vein, the disruption effects observed for grammatical sentences containing two negative elements could arise if the negative quantifier is still being integrated when *never* is encountered, forcing the parser to deal with two negative elements simultaneously. This interpretation suggests that the same incomplete encodings that could be ameliorating the online perception of unlicensed NPIs could also be responsible for deteriorating the perception of the sentences under investigation here. This would represent an illusion of ungrammaticality. Furthermore, these results provide evidence against the speculation that NPI illusions are the consequence of misrepresenting *ever* as its near neighbor *never*, given that continuations with *never* are judged as unacceptable in spite of their grammaticality. Together, these findings inform the landscape of hypotheses on NPI illusions and offer valuable insights into the complexity of multiple negations and the relation between processing difficulty and acceptability.

## Introduction

A central question in the study of sentence comprehension has to do with defining the role that grammatical information plays during the incremental interpretation of language. In this quest, the focus has been placed on studying the sensitivity that language users exhibit to grammatical contrasts during sentence processing. This sensitivity appears to be quite detailed, as instantiated by the skillful accuracy with which language users routinely detect grammatical anomalies both in online experiments and in offline judgments (for reviews, see Kaan, 2007; Phillips et al., 2011; Sprouse et al., 2013; Sprouse and Lau, 2013; Lewis and Phillips, 2015). The grammatical richness with which the language comprehension system seems to operate makes it even more interesting when the outputs of sentence processing do not converge with the constraints of the grammar. Misalignments between grammar and parsing provide a unique window into the principles that guide language comprehension, and their study has been a fruitful program in psycholinguistic research, giving way to numerous models and theories. Such grammar-parser discrepancies have been identified in a variety of structures and are explained by appealing to different grammatical and psychological principles. Without getting into the details of each specific case for reasons of space, the current mosaic of misalignments can be summarized attending to two criteria: first, whether they occur in grammatical or ungrammatical sentences; second, whether they are revealed in *fast* responses (observed in online processing tasks) or they also impact *slow* responses (observed in offline acceptability judgments).

Since its early days, linguistics has subscribed to the relatively uncontroversial view that grammatical sentences may be deemed unacceptable for reasons that are independent of grammatical theory (Chomsky, 1957). Some sentences are – almost – impossible to parse because their complexity exceeds the capacity of the system, leading to processing overload. This is the case of widely studied phenomena like multiple center embedding (e.g., Chomsky and Miller, 1963; Miller and Isard, 1964; Gibson, 1998) or strong garden path sentences (e.g., Bever, 1970; Frazier and Rayner, 1982; MacDonald et al., 1994), illustrated in (1) and (2), respectively.

(1) The patient who the nurse who the clinic had hired admitted met Jack.
(2) The horse raced past the barn fell.

Even though these sentences abide by the constraints of the grammar of English, it has long been known that most native speakers find them incomprehensible, exhibiting great difficulties in processing tasks and judging them as unacceptable in offline ratings. The opposite case can also be found, as certain ungrammatical configurations are sometimes processed and judged as if they were acceptable. So-called comparative illusions, illustrated in (3), are one of the most striking examples of this (Pullum, 2004; Wellwood et al., 2018). When native speakers are presented with sentences like (3), they remarkably judge them as both acceptable and meaningful; and only upon guided examination do they become aware of their ungrammaticality and semantic incoherence. A similar effect is observed when the multiple center-embedded sentences in (1) are presented to speakers with only two verbs instead of the required three, as shown in (4). Whereas the sentence is now ungrammatical, processing measurements and acceptability ratings improve when compared to its grammatical counterpart in (1) (Frazier, 1985; Gibson and Thomas, 1999; Gimenes et al., 2009; Häussler and Bader, 2015). This effect is sometimes referred to as the missing VP illusion. Comparative illusions and missing VP illusions are explained on the basis of different operations but display the same pattern of misalignment that opposes grammatical knowledge with online/offline responses.

(3) *More people have been to Russia than I have.
(4) *The patient who the nurse who the clinic had hired met Jack.

Although sentences like (3) are referred to as a *comparative illusions*, the label *grammatical illusion* is generally used to describe situations in which comprehenders fail to notice a grammatical error in processing tasks but clearly recognize the same sentences as unacceptable in offline judgments (Phillips et al., 2011; Lewis and Phillips, 2015). This is the case of agreement illusions, illustrated in (5) (Bock and Miller, 1991; Pearlmutter et al., 1999; Staub, 2009; Wagers et al., 2009) and negative polarity item illusions, illustrated in (6) and extensively covered in the next section. Despite the ungrammaticality of these examples, online processing measures indicate that the parser initially treats them as correct due to the presence of intervening elements: the plural *cabinets* in (5) and the negative quantifier *no* in (6). That is, grammatical illusions are typically described as discrepancies between fast (online) and slow (offline) responses, implying that online and offline measures of acceptability reflect qualitatively different aspects of linguistic behavior. In the general discussion, we will challenge such a neat view of grammatical illusions, as we hope to show that illusion-like patterns can emerge in the absence of a straightforward contrast between online and offline responses. Furthermore, even though grammatical illusions have attracted much interest in the past few years, the opposite phenomenon (i.e., illusions of ungrammaticality) is less often discussed. This project draws from research on negative polarity item (NPI) illusions in order to explore a candidate structure for illusions of ungrammaticality that illustrated by the grammatical sentence in (7) and explained in detail in section “The Current Investigation: Multiple Negation.”

(5) *The key to the cabinets are on the table.
(6) *The bills that no senators voted for have ever become law.
(7) The bills that no senators voted for have never become law.

The heterogeneous inventory of misalignments has motivated a debate about the role that grammatical information plays during sentence comprehension. This debate is embodied in the two systems/one-system divide (Lewis and Phillips, 2015). Proponents of a two system architecture (e.g., Townsend and Bever, 2001; Ferreira et al., 2002; Ferreira and Patson, 2007; Frank et al., 2012; Trotzke et al., 2013) argue that language comprehension and production are supported by a set of heuristic procedures that do not require speakers to build detailed grammatical information. Under this view, grammar is conceived of as a static body of knowledge that speakers can consult to verify the acceptability of sentences, and misalignments simply reflect the different outputs of these two systems. As Lewis and Phillips (2015) point out, this view is faced with the challenge of explaining how, in the majority of cases, comprehension and production actually exhibit grammatical richness and accuracy. By contrast, the strong convergence between grammar and parsing can be easily explained under a one-system view. In a one-system view, grammar and parsing are understood as forming a single cognitive system that serves the needs of comprehending and producing language (e.g., Phillips and Lewis, 2013; Embick and Poeppel, 2015; Lewis and Phillips, 2015; Mancini, 2018). In this architecture, grammar is an abstract description of the representations that the system builds. Instead of considering misalignments to be arbitrary failures, proponents of the one system view seek to understand the common profile of misalignments in order to systematically predict which linguistic computations will cause the system to err. In this vein, the present work takes NPI illusions as a starting point in order to explore a new and interesting instance of misalignment observed for grammatical sentences like (7). We start by discussing the specifics of NPI illusions that motivate this investigation.

### Negative Polarity Item Illusions

NPIs constitute a closed class of lexical items instantiated by words like *ever, any,* or *yet* that tend be used to strengthen the statements in which they appear (Kadmon and Landman, 1993). The heterogeneous nature of the contexts in which NPIs are licensed has motivated a wide range of theories within formal linguistics. These tend to capture the licensing conditions as an interaction of syntactic, semantic and pragmatic mechanisms (Barker, 1970, see Barker, 2018 for a recent review; Ladusaw, 1979; Linebarger, 1987; Krifka, 1995; Giannakidou, 1998, 2011). One of the most prominent licensing environments for NPIs is contexts that have some negative element^1^. For example, in (8a) the NPI *ever* is licensed by the presence of the negative quantifier *no* in subject position, while its absence in (8b/c) renders the sentences ungrammatical. Yet – as becomes apparent in the ungrammaticality of (8b) – mere linear precedence of the negative element is not enough: the NPI must occur in a position in which the negative quantifier is structurally accessible, a condition that is often explained as corresponding to overt c-command (Laka, 1994).

(8) a. **No** authors [that the critics recommended] have **ever** received acknowledgement for a best-selling novel.
b. *The authors [that **no** critics recommended] have **ever** received acknowledgement for a best-selling novel.
c. *The authors [that the critics recommended] have **ever** received acknowledgement for a best-selling novel.
  (Parker and Phillips, 2016)

The most interesting property of sentences like (8b) is that comprehenders often fail to notice their ungrammaticality because the presence of the negative quantifier in the relative clause reduces the effects of disruption observed for unlicensed NPIs, such as *ever* in (8c). Even though (8b) and (8c) are equally ungrammatical, processing experiments find (8b) to be parsed with much more ease than (8c). However, illusion effects do not always improve ungrammatical sentences like (8b) on a pair with perfectly grammatical ones. For example, in speeded acceptability tasks, NPI illusions arise as a three-way distinction^2^ among the conditions. Importantly, the interference generated by the negative quantifier seems to be only temporary. When participants are given enough time to judge the sentences, both (8b) and (8c) are recognized as unacceptable. This interference effect is known as an NPI illusion, a subtype of illusion of grammaticality. It is empirically robust across languages and measurements, such as speeded acceptability (German: Drenhaus et al., 2005; English: Parker and Phillips, 2016; de-Dios-Flores et al., 2017; Korean: Yun et al., 2018), self-paced reading (English: Xiang et al., 2013; Parker and Phillips, 2016), eye-tracking (German: Vasishth et al., 2008), or event-response potentials (German: Drenhaus et al., 2005; English: Xiang et al., 2009; Turkish: Yanilmaz and Drury, 2018).

Initial accounts of NPI illusions explore two different licensing routes debated in the grammar of NPIs as the source of the effect. On the one hand, Vasishth et al. (2008) propose that the interference effect arises as the consequence of retrieving the irrelevant licensor *no* due to partial feature match in a cue-based memory architecture (Lewis and Vasishth, 2005). This account rests on the assumption that NPI licensing involves establishing a direct item-to-item dependency between the NPI and a grammatical licensor using semantic (i.e., [+negative]) and syntactic (i.e., [+c-command]) cues. Thus, partial match with one of the two cues (i.e., [+negative]) would generate the acceptability illusion. However, it has been argued that NPIs can also be licensed pragmatically through negative inferences (Linebarger, 1987; Giannakidou, 2006). Building on this idea, Xiang et al. (2009, 2013) proposed instead that illusory licensing could be the result of generating negative inferences about the contrasting set of referents denoted by the relative clause in (8b), that is, *the authors that the critics recommended,* which would not have the predicated property (i.e., *receive and acknowledgment*). According to this proposal, these erroneous negative inferences could produce the licensing illusion. While these two accounts appeal to different grammatical resources available to license NPIs (syntactic-semantic vs. pragmatic), they both explain the intrusion effect by the misapplication of the licensing mechanisms activated when encountering the NPI. Accordingly, the two views predict, in broad terms, that illusions should generalize to different items and configurations whenever an NPI has to be licensed.

Nonetheless, a more recent investigation by Parker and Phillips (2016) has provided compelling experimental and modeling evidence that the configurations that yield NPI illusions are more restricted than it was initially thought. In a series of experiments, they demonstrate that the intrusion effect can be turned off by increasing the distance between the NPI and the illicit licensor as in (9)^3^ or (10). This behavior is not predicted by previous accounts. Parker and Phillips (2016) argue that the *on/off* behavior of NPI illusions points to changes in the status of the encoding that is probed for licensing at the point of dependency formation, emphasizing the idea that linguistic encodings are not stable but, rather, take some time to complete. Consequently, NPI illusions reflect access to intermediate stages of the encoding process. When the NPI is checked against the licensing context soon after the relative clause has been encountered, the irrelevant negation may be transparently accessible to spuriously license the NPI. However, when the encoding of the licensing context is accessed at a later point in time, as in (9) and (10), the material inside the relative clause is – presumably – fully encoded and no longer accessible for licensing. This proposal will be referred to as the *changing encodings hypothesis*. Even though it focuses on memory encoding mechanisms rather than retrieval ones, this view is presented as compatible with a cue-based parsing architecture. Putting together ideas from Vasishth et al.’s (2008) proposal with other parsing models that do assume that the format of representations changes over time (e.g., tensor-product variable bindings or vector-based models), Parker and Phillips (2016) speculate that NPI illusions could result from a two-stage representation building process: during a first stage, individual feature values – such as negation – are thought to be transparently accessible giving way to partial match interference. Thus, the licensing illusion could occur during this first stage. In the second stage, individual features are thought to be bound together into a distributed representation, and they could no longer be independently evaluated, blocking illusions to happen. Such an explanation can account for the presence of interference in sentences like (8a) and the absence of it in sentences like (9) and (10).

(9) The authors [that **no** critics recommended] have received **any** acknowledgement for a best-selling novel.
(10) The authors [that **no** editors recommended] have, as the editor mentioned, **ever** received a pay raise.

An alternative speculation about NPI illusions, which will be referred to as the *ever-never confusability hypothesis*, has not been explicitly maintained or experimentally tested before, but it is briefly discussed by Parker and Phillips (2016, pp. 227–228). This proposal hypothesizes that a confusion between *ever* and *never* could be behind the improved perception of NPI illusion sentences. Such a confusion is thought to be facilitated by the orthographic and phonological similarities of the two words, and crucially, because substituting *ever* with *never* would provide a grammatical continuation for NPI sentences like (8b). A process of this sort can be conceptualized under a noisy-channel architecture of sentence comprehension (Levy, 2008a,b; Levy et al., 2009; Gibson et al., 2013). Noisy-channel models assume that retaining each individual word in short-term memory introduces a degree of uncertainty about the previous input. When processing problems are encountered, this uncertainty gives rise to the possibility of misrepresenting previous words in the sentence in cases in which a near neighbor would allow a more probable structure and/or repair an error in the input. For the case of NPI illusions, uncertainty about the input is expected to increase when comprehenders encounter an unlicensed NPI, causing *ever* to be misrepresented in a proportion of cases as its near neighbor *never*, repairing the ungrammaticality^4^. But, why would comprehenders misrepresent the input for sentences with an irrelevant licensor (8b) and not for sentences with no licensor at all (8c)? A possible explanation is that *never* is actually a more plausible continuation for sentences containing the negative quantifier in the relative clause than it is for sentences without it. If we take the examples in (8), it is easier to conceive a situation in which a set of authors have never received acknowledgement when they were not recommended by the critics (8b), than when they were recommended by the critics (8c). Consequently, *ever* could be more often misrepresented as *never* in (8b) than in (8c), explaining the improved perception of NPI illusion sentences. The present investigation explores the *changing encodings* and the *ever-never confusability hypothesis* by examining the processing and acceptability of sentences in which the NPI *ever* was substituted by the negative adverb *never.* Sections “The Current Investigation: Multiple Negation” to “Predictions: Relating Multiple Negation to Negative Polarity Item Illusions” present the details of the experimental design and the specific predictions on which it is articulated.

### The Current Investigation: Multiple Negation

The experiments presented here make use of different configurations of negative elements as a means to test two contrasting predictions inspired by previous accounts of NPI illusions. For this purpose, this investigation focuses on grammatical sentences, which vary the presence and structural location of the negative determiner *no* with respect to the adverb *never.* This manipulation results in the three contrasts shown in Table 1: single negation (condition A), multiple negation (condition B), and double negation (condition C). The main objective of the project is to study the processing and acceptability of multiple negation sentences (condition B). In these sentences, the negative adverb *never* is linearly preceded by a structurally inaccessible negation, the quantifier *no* inside the relative clause. Multiple negation sentences could be considered the opposite configuration of NPI illusions in that when the NPI *ever* is substituted by *never,* they become grammatical strings. More importantly, as noted in Huddleston and Pullum (2002), the words *ever* and *never* are semantically and etymologically related: both elements express a quantification in terms of frequency or temporal location, despite having different syntactic distributions. While the NPI *ever* adds a quantificational force to an already negated statement, *never* expresses the negative and quantificational forces at the same time. Thus, sentences like *No authors have ever received acknowledgement* and *The authors have never received acknowledgement* are roughly equivalent.

**Table 1:** Sample set of experimental conditions.

**A. Single negation**	**The** authors [that **the** critics recommended] have **never** received acknowledgment for a best-selling novel.
**B. Multiple negation**	**The** authors [that **no** critics recommended] have **never** received acknowledgment for a best-selling novel.
**C. Double negation**	**No** authors [that **the** critics recommended] have **never** received acknowledgment for a best-selling novel.

In order to study the processing and acceptability of multiple negation sentences, they will be compared to single negation and double negation sentences using three tasks: a speeded acceptability task (Experiment 1), a self-paced reading task (Experiment 2), and an untimed acceptability judgment (Experiment 3). The first two experiments are devised to tap into the online/fast processing of these structures, while Experiment 3 focuses on speakers’ offline/slow perception of acceptability. Importantly, the three experimental conditions tested here are grammatical in English, even though they vary in their syntactic and semantic complexity. For the purpose of this investigation, single negation sentences are taken to be the simplest of the three and serve as an unproblematic baseline for comparison. On the other end, instances of double negation are assumed to generate processing and acceptability problems, and are used as some sort of “unacceptable” or degraded baseline. These initial assumptions are based on previous linguistic considerations, which are reviewed in the next section.

### Some Notes on Negation

All natural languages express negation (Horn, 2001). Yet, in spite of the high frequency with which negative expressions appear in routine language use, negative statements have been related to an increase in processing effort when compared to equivalent affirmative statements (Wason, 1961; Fischler et al., 1983; Carpenter et al., 1999; Kaup et al., 2006; Herbert and Kübler, 2011). There is vast cross-linguistic variation on how the operation of negation can be carried out with regard to “the position of negative elements, the form of negative elements and the interpretation of sentences that consist of multiple negative elements” (Zeijlstra, 2007, p. 498). In English, negation can be marked by words (e.g., *no, not* or *never*) or by affixes (e.g., -*n’t* or *in-*). For instance, in the single negation condition in Table 1, the negative adverb *never* expresses sentential negation. As regards the presence of more than one negative element in a sentence, one can often find sentences composed of two clauses that are independently negated. This case is illustrated by multiple negation sentences. Zeijlstra (2004, p. 58) points out that these sentences should not be considered as double negation because “two propositions are negated one, but no proposition is negated twice”. To avoid confusion, the label *multiple negation* will only be used to refer to these sentences.

Furthermore, when two negative expressions interact in the same clause, they can form two types of dependencies: negative concord or double negation. Negative concord dependencies are observed in languages in which the presence of two negative elements is interpreted as a single semantic negation (e.g., Spanish, Italian, or African-American Language). Conversely, Standard English is commonly classified as a double negation language, in which each negative marker contributes an independent semantic negation. In double negation languages, the two negative elements cancel each other out yielding an affirmative interpretation as a result (Horn, 2001, 2010; de Swart, 2010; Puskás, 2012 i.a.). This is exemplified by the double negation sentence in Table 1, which could be paraphrased as *All the authors that the critics recommended have received acknowledgement for a best-selling novel at least once*. Double negation dependencies entail complex operations in terms of the syntactic, semantic and prosodic marks that are needed. For instance, it has been found that the use of specific contradictory intonational contour and denial gesture features are crucial for the felicitous interpretation of double negation dependencies in oral comprehension tasks (Espinal and Prieto, 2011; Prieto et al., 2013). In written format, a corpus study by Larrivée (2016) described that double negation dependencies are generally triggered in restricted information-structure configurations in which a discourse-old negative statement is being denied by the second negation. Due to its complexity, double negation dependencies are assumed to engage in greater processing cost than negative concord dependencies or single negation (Corblin, 1996). Unfortunately, the psycholinguistic studies on the processing of double negatives are very scarce.

Using a sentence verification task, Sherman (1976) tested multiple combinations of negative elements (from 1 up to 5 negative markers).^5^ His results clearly show that the presence of two negative elements in a sentence considerably increases comprehension time and error rates. Another study by Schiller et al. (2017) used the Event-Related Potential technique in order to study configurations that combine verbal negation and affixal negation (e.g., *not impossible*). Their findings show that, at least for these simpler combinations, the processing disruptions associated with double negation can be overruled by discourse contexts that clearly evoke negative expectations. Putting the evidence from these previous studies together, it appears that instances of double negation seem to present parsing difficulties when there are not explicit pragmatic cues that help predict the double negative dependency. Along these lines, Blanchette (2013, see also Blanchette et al., 2018) maintains that speakers of Standard English tend to interpret instances of double negation as negative concord dependencies when they are encountered in the absence of the relevant cues. This claim is also supported by experimental evidence provided by Thornton et al. (2016), showing that young children acquiring Standard English initially perceive double negation configurations as forming negative concord dependencies. Taking all this into account, our starting assumption is that the double negation dependencies used here (i.e., condition C, Table 1) will generate strong processing difficulties and will be deemed unlicensed when encountered in isolation. By contrast, single negation (i.e., condition A, Table 1) is expected to be processed with ease and to be recognized as acceptable. These assumptions are set to test in the experiments that follow, and their endorsement is essential in order to interpret them as baselines. Before moving into the experimental evidence, the next section discusses the specific predictions that relate multiple negation sentences to NPI illusions.

### Predictions: Relating Multiple Negation to Negative Polarity Item Illusions

Given that the grammar of *never* is not constrained by the licensing conditions that affect NPIs, this investigation does not address explanations of NPI illusions that invoke the faulty implementation of NPI-specific licensing mechanisms (Vasishth et al., 2008; Xiang et al., 2009, 2013). The interest of this project lies, instead, on exploring two conflicting predictions that can be extracted from the *ever-never confusability* and the *changing encodings* hypotheses. Multiple negation sentences provide a ground for testing these two proposals because they predict opposite patterns of results.

On the one hand, if NPI illusions are the result of misrepresenting *ever* as *never*, multiple negation sentences display the precise configuration that is assumed to rescue unlicensed NPIs. In a noisy-channel architecture, comprehenders would be likely to misinterpret *ever* as *never* in cases in which *never* provides a more plausible and/or natural sentence. Here, a correspondence is assumed between plausibility and grammaticality, as sentences containing *never* are thought to be more plausible because they provide a grammatical and meaningful continuation. This hypothesis predicts that multiple negation sentences should be recognized as acceptable by native speakers of English and should be parsed with ease. If multiple negation sentences are perceived as acceptable, they are expected to pattern closer to single negation sentences (which are assumed to be perceived as acceptable) than to double negation sentences (which are assumed to generate problems). Importantly, these predictions result from our understanding of the *ever-never confusability hypothesis* within a noisy-channel architecture, as this proposal had never been explicitly articulated until now. In our view, an explanation that appeals to a misrepresentation of *ever* as *never* is in conflict with multiple negation sentences generating strong processing or acceptability problems; because such a misrepresentation is only motivated when it leads the parser into an acceptable and unproblematic structure. The *ever-never confusability hypothesis* – or any other account of NPI illusions – does not predict sentences like (8b) to be perceived on a pair with perfectly grammatical ones. Yet, this hypothesis rests on the assumption that similar sentences containing *never* should be generally processed and recognized as acceptable.

On the other hand, the *changing encodings hypothesis* put forth by Parker and Phillips (2016) predicts the opposite outcome. Under this view, NPI illusions are the result of accessing incomplete computations of the material inside the relative clause that includes the quantifier *no*, facilitating a dependency between the spurious licensor and *ever*. The negative quantifier could be retrieved as a licensor – possibly in a cue-based procedure – because its individual features can be transparently accessible in early stages. Accordingly, the same intermediate stage computations are expected to be in place in multiple negation sentences up to the point when participants reach *never*. If the negation inside the relative clause has not been bounded into a distributed representation when *never* is encountered, the parser may experience problems in having to integrate two negative elements simultaneously. This cost is predicted to manifest as a disruption in reading times in multiple negation sentences relative to single negation. Importantly, the content that precedes *never* in multiple negation sentences is the same that precedes *ever* in Parker and Phillips’ (2016) illusion configurations. Thus, finding similar interference effects in online tasks could indicate that the same incomplete encodings that temporarily improve the perception of ungrammatical NPI configurations are responsible for hampering the comprehension of grammatical multiple negation sentences. A possible speculation is that the disruption predicted in multiple negation sentences could index the parser’s difficulties evaluating a double negation dependency between *no* and *never*. Assuming that double negative dependencies are problematic in the absence of enough contextual cues, entertaining an illusory double negation dependency is expected to generate similar effects to those expected in actual double negation sentences.

In sum, the fundamental question that this research aims to answer is whether multiple negation sentences are processed and judged closer to single negation sentences (which are expected to be processed without any problems), or to double negation sentences (which are expected to generate strong disruptions). Moreover, although this investigation takes NPI illusions as a point of departure, we hope that it will also provide insights into the processing and grammatical status of double negation dependencies; a phenomenon that still remains poorly explored from a psycholinguistic perspective.

## Experiment 1: Speeded Acceptability Judgment

Experiment 1 used the speeded acceptability technique to investigate whether the perception of grammatical sentences containing two negation markers is degraded for sentences in which these negative elements do not engage in a negative dependency. Speeded acceptability judgments are generally considered an *online* technique because the limited amount of time provided to respond forces participants to operate on fast and unconscious intuitions of grammaticality. They have been reliably used as a time-sensitive measure to test NPI illusion configurations (e.g., Drenhaus et al., 2005; Parker and Phillips, 2016; de-Dios-Flores et al., 2017; Muller et al., 2019).

### Participants

Twenty-eight native speakers of English (19 female, mean age 20 y/o) participated in this experiment. They were recruited through the University of Maryland’s participant database. Participation was compensated with a credit in an introductory linguistics course or, alternatively, with $10. The speeded acceptability task was administered together with another unrelated experiment as part of a 1-h testing session. All the participants in this, and the following experiments provided informed consent and were naïve to the purpose of the experiment. They were also screened for native speaker abilities through a short questionnaire that tested constraints on tense, modality, morphology, ellipsis, and syntactic islands. In order to participate in the experiments, the candidates were required to pass the test with a minimum of 7/9.

### Materials

The experimental materials consisted of 36 sets of three items like those in Table 1. These were adapted from the stimuli used in Parker and Phillips (2016) by solely substituting the NPI *ever* by the negative adverb *never*. The experimental conditions were counterbalanced in three lists using a Latin Square design, together with 72-filler sentences of similar internal structure, length and complexity. Each list had a total of 108 items, and participants were randomly assigned to one of the three lists. Grammaticality was balanced so that half of the sentences were ungrammatical. This ensured that the initial probabilities of providing a *yes* or a *no* answer were equal across the task. For this purpose, double negation sentences (condition C) were counted as ungrammatical. To achieve a 1:1 grammatical-to-ungrammatical ratio, 42 fillers contained ungrammaticalities. The grammatical violations introduced included preposition usage, number agreement, verbal morphology and pronoun-antecedent mismatches. During the delivery of the instructions, participants were asked to complete six practice items to ensure that they had understood the procedure.

### Procedure

The stimuli for this speeded acceptability task were presented on a desktop PC using Ibex (Drummond, 2013). Each sentence was displayed word by word at a rate of 400 ms per word, in the center of the screen, using the rapid serial visual presentation (RSVP) paradigm. At the end of each sentence, a response screen appeared and participants were asked to provide a *yes*/*no* button press judgment in a maximum time of 2 s. When participants failed to provide the judgment in time, a message indicated that they were too slow. Participants were instructed to read the sentences carefully and judge whether they came across as well-formed English. They only received feedback for the first two practice items. All participants were tested on the same computer. The task lasted for approximately 30 min, and the order of presentation for experimental and filler sentences was randomized for each participant.

### Analysis

The *yes/no* responses collected were analyzed using a generalized linear mixed model for binomial distributions (also known as logistic mixed model; Baayen et al., 2008; Jaeger, 2008). A maximal model with a fully specified random effects structure was initially built. This model included the experimental conditions as fixed effects and by-participants and by-items random intercepts and slopes for the experimental conditions. Yet, this model failed to converge and had to be reduced to a model with random intercepts but no slopes. This was the maximally converging model. As noted in Barr et al. (2013, pp. 23–24), categorical data tend to pose more difficulties for maximal models to converge. For this and the following two experiments, the contrasts among the three experimental conditions were obtained as follows: first, condition A (single negation) was used as the reference level of the intercept in order to obtain the contrasts between A and B (multiple negation) and A and C (double negation). Then, the contrasts between B and C were obtained by setting B as the intercept. All the analyses reported for this and the following experiments were carried out using R, an open-source programming environment for statistical computing (R Development Core Team, 2014). Specifically, the models were estimated using the package *lme4* for linear mixed effects models (Bates et al., 2015). Following Gelman and Hill (2006), an effect was considered statistically significant at the *p* > 0.05 level when the absolute *z* value was above 2.

### Results

**Figure 1:**
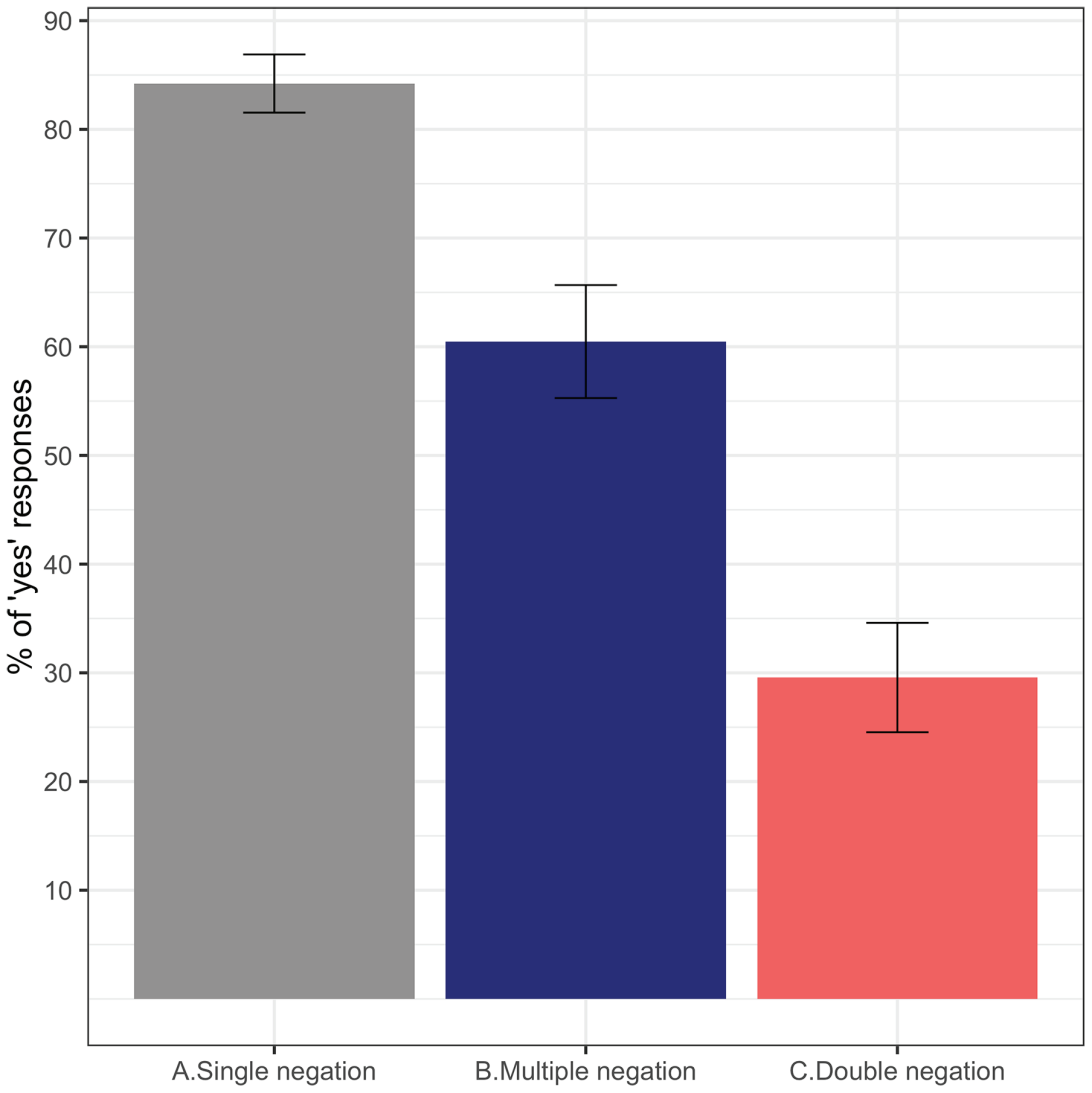
Average percentage of “YES” responses for the experimental conditions aggregated by participant (Experiment 1). Error bars indicate standard error of the mean.

Figure 1 shows the average percentage of *yes* responses to each of the three experimental conditions. Sentences containing a single negation (condition A) were judged as acceptable in most cases (above 80% acceptance). The presence of two negations significantly reduced the perception of acceptability for both multiple negation (A vs. B: $\overset{\wedge }{\beta }$ = −1.48, SE = 0.21, *z* = −7.06) and double negation sentences (A vs. C: $\overset{\wedge }{\beta }$ = −3.05, SE = 0.23, *z* = −13.35). Nonetheless, the decrease in acceptability was less acute when the two negations appeared in different clauses (condition B, above 60% acceptance) than for traditional double negatives (condition C, below 30% acceptance). This contrast was statistically significant (B vs. C: $\overset{\wedge }{\beta }$ = −1.57, SE = 0.19, *z* = −8.31).

### Discussion

This experiment tested the impact of different negation configurations on fast perceptions of acceptability using a processing demand task. Based on linguistic and psycholinguistic considerations, it was initially assumed that single negation sentences would be unproblematic for native speakers of English, and that double negation sentences would possibly be deemed unacceptable in the absence of the appropriate licensing context. These assumptions are borne out in the results. Importantly, the perception of acceptability of grammatical multiple negation sentences is penalized, although there is a significant three-way distinction among the conditions: single negation sentences are accepted in the vast majority of cases, multiple negation sentences exhibit a lower but still greater proportion of *yes* over *no* responses, and double negation sentences are rejected in the majority of cases. That is, the perceived ungrammaticality increased when *no* c-commanded *never* than when it did not. The acceptability contrast between single negation and multiple negation sentences cannot be attributed to constraints of the grammar or other linguistic considerations, as both sentences are perfectly grammatical. Instead, it points to a processing problem as the source of the effect. These results are interpreted as initial evidence that the presence of a structurally inaccessible negative quantifier *no* interferes with the integration of the adverb *never* in the main clause. The pattern of results bears significant resemblance to the picture that arises in speeded acceptability studies of NPI illusions (e.g., Drenhaus et al., 2005; Parker and Phillips, 2016; de-Dios-Flores et al., 2017; Muller et al., 2019). In these studies, an illusion of grammaticality is identified with higher acceptance rates for unlicensed NPIs in sentences with *no* inside the relative clause (e.g., *The authors [that no critics recommended] have ever received acknowledgement for a best-selling novel*), compared to sentences without it. The pattern found in this experiment is the exact opposite: lower acceptance rates for grammatical sentences with *no* inside the relative clause (i.e., multiple negation) than for similar grammatical sentences without it (i.e., single negation). In other words, while the intrusive *no* ameliorates the perception of *ever* in ungrammatical sentences, it seems to deteriorate the perception of *never* in grammatical sentences.

Speeded acceptability tasks gather information about the participants’ overall initial perception of acceptability, and they have been proved to be a reliable technique uncovering grammatical illusions. Even though participants respond once the full sentence has been presented, the proportion of correct judgments is generally assumed to relate to processing operations due to the time pressure under which these are elicited. However, as Vasishth et al. (2008, pp. 696–697) point out, “the source of the judgment itself is presumably a decision process that takes as input the products of (possibly partially) completed online processing.” Thus, this task does not allow us to ascertain which are the specific sentence regions that generate this behavior or to disentangle sentence comprehension mechanisms from other processes that affect end-of-sentence decisions. The next experiment was designed to delve deeper into the source of the interference effect.

## Experiment 2: Self-Paced Reading

This experiment uses a self-paced reading task in order to study the online processing of the sentences under investigation. This method provides access to moment-by-moment processing during the automatic integration of each sentence word and the difficulty generated by it. In light of Experiment 1, the integration of *never* is expected to take place without problems in single negation sentences and to generate strong processing disruptions in double negation sentences. With regard to the critical condition (i.e., multiple negation sentences), if the presence of the negative quantifier *no* in the relative clause interferes with the integration of *never* in the main clause, longer reading times are expected at the point of *never* in multiple negation sentences relative to single negation sentences.

### Participants

The participants in this experiment were 36 native speakers of English (30 female, mean age 24 y/o) who were recruited in the area of Santiago de Compostela. All of them were pursuing or had just finished university education (BA or MA) in different disciplines in the USA and were serving as high school language assistants as part of a 1-year exchange program funded by the Galician Regional Ministry of Education^6^. Special care was taken to ensure that none of the participants had spent more than 48 months outside an English-speaking country across their entire life. Their participation in the study was voluntary.

### Materials

The experimental materials used in this task were the same as in Experiment 1 (see Table 1). The three conditions were counterbalanced in three lists together with a grammatical version of the same 72-filler sentences. The ratio of ungrammatical-to-grammatical sentences was reduced in order to prevent participants from developing unnatural reading strategies. In order to ensure that participants were reading for comprehension, all the experimental and filler sentences were followed by a *yes*/*no* question. These comprehension questions addressed pieces of information located in different parts of the sentences. This way, participants were forced to pay equal attention to all the sentence regions. The comprehension questions for the experimental items were never related to the negated material and the probability of providing a positive or a negative answer was balanced. During the delivery of the instructions, participants were asked to complete four practice items to ensure that they had understood the procedure.

### Procedure

The task was implemented in Inquisit 4 (Millisecond Software, 2015) using the non-cumulative word-by-word moving window version of the self-paced reading procedure (Just et al., 1982). In this version of the task, participants are presented with the entire sentence on the screen with each word masked by dashes and separated by spaces. When the predefined key is pressed (the space bar in this case), the first word is revealed. When the space bar is pressed one more time, the second word appears and the first word is re-masked. By collecting the time elapsed between bar-presses, this task allows us to measure the time spent in each word. Participants were instructed to keep their fingers on the selected keys (i.e., the space bar and *yes/no* keys) for the entire session. This way, they could move forward easily at their own pace and answer the questions as accurately and as fast as possible. They received on screen feedback for both wrong and right answers – the word “right” was displayed for 1,000 ms when the response was correct, and the word “wrong” was displayed for 2,000 ms when the response was incorrect. All participants were tested using the same computer. The task lasted for approximately 35 min, and the order of presentation of experimental and filler sentences was randomized for each participant.

### Analysis

The acceptance threshold for accuracy in the questions was set to 80% to ensure that the final sample only contained participants that were reading for comprehension. No participant had to be excluded from the analysis due to poor performance. Unrealistic reading times were first deleted following standard practices in the self-paced reading literature (see for example Hofmeister, 2011; Nicenboim et al., 2016). These included RTs above 2,500 ms and below 100 ms, which are possibly the product of spurious delays or erroneous button presses that might obscure the initial stages of model fitting (Baayen, 2008). This procedure resulted in the exclusion of 0.85% of the data across all sentence regions and 0.23% of the data in the regions of interest. Subsequently, the remaining reading times were log-transformed in order to reduce non-normality. Average RTs for the experimental conditions were then compared in four regions of interest: the auxiliary verb before *never*, which signals the end of the relative clause; the negative adverb *never,* which is the critical point at which the different negation configurations are established; and the two next spillover words. These reading times were analyzed using a linear mixed effects model.

Following the same model building procedure as in Experiment 1, the RTs in the four regions of interest were analyzed using the maximally converging model (Barr et al., 2013). The maximal model included the experimental conditions as fixed effects and by-participant and by-item random intercepts and slopes. This model was applied in the pre-critical region, the critical region (*never*), and the first spillover region. In the second spillover region, the maximal model had to be reduced due to convergence problems. This reduced model included by-participant and by-item random intercepts but no slopes. The accuracy for the comprehension questions in the experimental trials was also analyzed. This was done by means of a generalized linear mixed model for binomial distributions (Jaeger, 2008). The maximally converging model included fixed effects for the experimental conditions and only random intercepts for participants and items. An effect was considered to be statistically significant at the level of *p* < 0.05 when the absolute *t or* z value was above 2 (Gelman and Hill, 2006; Baayen et al., 2008).

### Results

**Figure 2:**
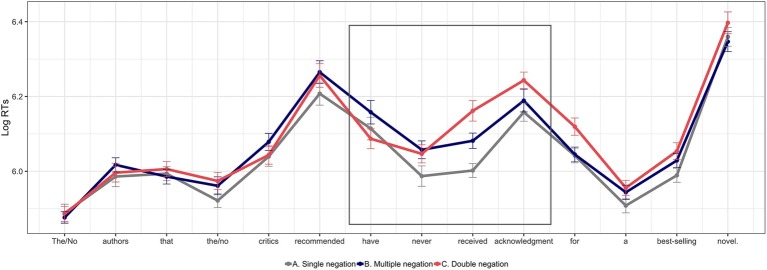
Average word-by-word reading times for the experimental conditions aggregated by participant (Experiment 2). Error bars indicate standard error of the mean. The regions of interest are contained within the square.

Figure 2 shows the average word-by-word reading times in log-milliseconds for the three experimental conditions in all the sentence regions. The four regions of interest are highlighted inside a square. The model results for the pre-critical region (the auxiliary *have*) show a significant effect of multiple negation when compared with double negation (B vs. C: $\overset{\wedge }{\beta }$ = −0.07, SE = 0.03, *t* = −2.20). This region was read slower for multiple negation sentences (condition B) than for double negation sentences (condition C). The contrast with single negation (condition A) was not statistically significant (A vs. B: $\overset{\wedge }{\beta }$ = 0.04, SE = 0.03, *t* = 1.30), even though it follows a similar trend. The results for the adverb *never* (the critical region) show that sentences with two negation markers (multiple and double negation sentences) are read more slowly than sentences in which *never* was the only negative element –single negation sentences – (A vs. B: $\overset{\wedge }{\beta }$ = 0.07, SE = 0.03, *t* = 2.21; A vs. C: $\overset{\wedge }{\beta }$ = 0.06, SE = 0.03, *t* = 2.19). Furthermore, no differences are observed between multiple negation and double negation sentences in the *never* region (B vs. C: $\overset{\wedge }{\beta }$ = −0.01, SE = 0.03, *t* = −0.37). The slow-down for sentences with two negations extends to the first spillover region in a three-way contrast: single negation sentences were the fastest of the three (A vs. B: $\overset{\wedge }{\beta }$ = −0.08, SE = 0.03, *t* = 3.02; A vs. C: $\overset{\wedge }{\beta }$ = 0.16, SE = 0.03, *t* = 4.95). Furthermore, double negation sentences displayed a more pronounced slowdown than multiple negation sentences (B vs. C: $\overset{\wedge }{\beta }$ = −0.08, SE = 0.03, *t* = 2.88). In the second spillover region, there was a significant effect of double negation, reflecting slower reading times relative to both single negation and multiple negation sentences (A vs. C: $\overset{\wedge }{\beta }$ = 0.08, SE = 0.03, *t* = 3.24; B vs. C: $\overset{\wedge }{\beta }$ = 0.05, SE = 0.03, *t* = 2.12). No differences are observed between single negation sentences and multiple negation sentences in this second spillover region (A vs. B: $\overset{\wedge }{\beta }$ = 0.03, SE = 0.03, *t* = 1.11). Average accuracy for the comprehension questions in the experimental items was 94% (condition A: 96%, condition B: 96%, condition C: 91%). The results from the logistic regression indicate a significant decrease in accuracy for double negation sentences when compared to the other two conditions (A vs. B: $\overset{\wedge }{\beta }$ = −0.02, SE = 0.38, *z* = 0.95; A vs. C: $\overset{\wedge }{\beta }$ = −0.95, SE = 0.32, *z* = −2.93; B vs. C: $\overset{\wedge }{\beta }$ = −0.92, SE = 0.32, *z* = −2.89).

### Discussion

This experiment used the self-paced reading technique to investigate whether the interference effects found in Experiment 1 reflect difficulties in the integration of *never* during the incremental processing of multiple negation sentences. Before examining the results for this critical condition, it is important to note that the reading times for the baseline conditions are aligned with the initial predictions as well as with the results from Experiment 1: single negation sentences are read the fastest of the three, and double negation dependencies did not only impact reading times but also caused a reduction in comprehension question accuracy. This drop in accuracy could be initially surprising because the comprehension questions never targeted information related to the negations and were the same in the three experimental conditions. A plausible explanation for this behavior is that the confusion generated when participants tried to interpret double negated sentences prevented them from paying enough attention to the rest of the contents. The reading times for multiple negation sentences in the regions of interest seem to confirm the intuition that the decrease in acceptance observed in Experiment 1 could arise from the difficulty of integrating *never* when it is preceded by the embedded negative quantifier *no*. Importantly, when comprehenders reach the negative adverb, the reading times for multiple negation and double negation sentences are on a par. The disruption observed for sentences with two negations spills over the sentence regions following *never*, even though participants recover earlier in multiple negation than in double negation conditions. The pattern of results found in this experiment seems initially incompatible with the hypothesis that NPI illusions arise due to a misrepresentation of *ever* as *never.* The *ever/never confusability hypothesis* rests on the assumption that sentences with *never* are both an acceptable and natural continuation, but multiple negation sentences are shown to create processing problems. Such problems are not expected if multiple negation sentences represent the configuration that is thought to ameliorate NPI illusions. The fact that the RTs at the critical region show the same slowdown in both multiple and double negation sentences is particularly relevant because this is the region in which unlicensed NPIs such as (8b) display the strongest facilitation effects. Nonetheless, it is difficult to map the RTs in this experiment to those in classic NPI illusion sentences because of the different baselines used. The offline ratings from the next experiment will hopefully clarify the perceived status of multiple negation sentences.

One potential concern with these results is that the reading times for multiple negation sentences are slower than the other two conditions in the region preceding *never.* Up to this point, the sentences used here are identical to those in Parker and Phillips’ (2016) self-paced reading task (Experiment 3 in their work), but they do not observe any significant effects in the pre-critical region. In spite of the lack of statistical contrasts, Parker and Phillips’ (2016) data display a similar trend: the auxiliary *have* is read slower in sentences containing *no* inside the relative clause. Given that our sample contained 50% more participants than Parker and Phillips’ experiment (*n* = 24), we believe that the two pre-critical effects could be qualitatively similar, but their study lacked the necessary power to detect the contrast. It is also possible that the effect found at the pre-critical region is stronger in our data as a consequence of the experimental manipulation. The auxiliary *have* provides a structural cue that signals the end of the relative clause, and it is always followed by the critical region – *ever* in NPI illusions and *never* in these sentences. However, in the study by Parker and Phillips, the presence of the negative quantifier facilitated the integration of *ever,* while here, its presence seems to hamper the integration of *never.* As the experiment unfolds, the problems associated with the different configurations of negative elements could have made both the quantifier *no* and the adverb *never* more salient in our experiment, and thus, participants could be placing more resources to process the negative quantifier inside the relative clause before reaching the negative adverb. Such an effect is predicted to surface as a slowdown only in multiple negation sentences, as it is the only condition that displays an embedded negation. As suggested by an anonymous reviewer, this conjecture predicts the effect to grow across the experiment, and thus, it can be investigated by modeling the interaction with trial order. However, the results from a post-hoc analysis clearly showed the opposite: the contrast between multiple negation sentences and the other two conditions was the strongest during the first trials and shrank dramatically across the task^7^, discarding this additional possibility. In sum, the fact that the pre-critical effects only arise in multiple negation sentences is interpreted as evidence that at least some aspects of the embedded negation are still being integrated when participants reach the auxiliary *have.* That is, the difficulty associated with the integration of the negative quantifier seems to spill over outside of the relative clause.

If the quantifier *no* has not been fully encoded when comprehenders reach *never,* the slower reading times observed for multiple negation sentences could reflect the difficulties of the parser when trying to integrate two active negative elements. In order to support this interpretation, it is essential to establish whether the contrast observed at the critical and post-critical regions between single negation and multiple negation sentences is not simply the consequence of the spillover effect from the embedded negation. In other words, that there is some additional processing difficulty specifically triggered by *never.* To explore this issue, we calculated Cohen’s delta (*d*) statistic (Cohen, 1988) for the contrast between single and multiple negation sentences in the pre-critical, the critical and the post-critical regions. The results show that whereas the effect size in the pre-critical region is quite small (*d* = 0.12), the effect size in the critical region is almost three times bigger (*d* = 0.34), and even more so in the post-critical region (*d* = 0.51). The fact that the effect grows when *never* is encountered represents evidence that the negative adverb contributes its own source of processing difficulty, and thus, that the disruption observed at the critical and post-critical regions could be reflecting the combined difficulty of integrating the two negative elements. Such interpretation of the results aligns with Parker and Phillips’ (2016) hypothesis that NPI illusions arise as a consequence of unstable encodings available when the NPI is being licensed. Under this hypothesis, the slow reading times observed at the negative adverb would reflect the difficulties of the parser to integrate *never* in the context of a previous negative element. As it was speculated in the predictions section, such a disruption could be indexing initial attempts of the parser to entertain a temporary double negation dependency between *never* and *no.* This idea is motivated by the fact that the RTs at the critical region are equally slow for multiple and double negation sentences. The crucial difference between these two conditions is that, in multiple negation sentences, this dependency is not structurally supported, and this could be interpreted as an illusion of ungrammaticality. Two facts seem to support the idea that such an illusory double negation dependency could just be temporarily entertained. First, participants recover earlier from the disruption produced in multiple negation sentences than in double negation sentences. Second, this interference does not seem to have interpretive consequences, inasmuch as comprehension question accuracy is not reduced in multiple negation sentences. The general discussion delves deeper into this issue.

Together, Experiments 1 and 2 provide clear evidence that the negative quantifier *no* inside the relative clause interferes with the online integration of *never* in the main clause. In line with Parker and Phillips’ (2016) account of NPI illusions, this interference effect is expected to arise during early parsing stages in which the encodings of the material in the relative clause, and the quantifier *no* in particular, have not been fully computed. Under the assumption that comprehenders only experience an illusion of ungrammaticality in online tasks, native speakers of English are expected to recognize multiple negation sentences as acceptable when given ample time. The objective of Experiment 3 is to test the offline perception of acceptability of sentences under investigation.

## Experiment 3: Offline Acceptability Rating

This section presents the results from an offline acceptability judgment (Cowart, 1997). As explained above, acceptability measures will contribute to understand the causes and interpretation of the disruption observed for multiple negation sentences in Experiments 1 and 2. In addition, these untimed ratings will further corroborate the grammatical status of the baseline conditions.

### Participants

Twenty-four US-based native speakers of English (6 female, mean age 35 y/o) participated in this experiment. All participants provided informed consent and they received $3 as compensation. The experiment lasted approximately 20 min. Participants were recruited using Amazon’s Mechanical Turk (AMT; https://aws.amazon.com/mturk). AMT is a crowdsourcing web-service through which institutions and companies can recruit participants for human intelligence tasks. Its use in the fields of linguistics and psychology has increased in recent years, and several studies have already validated its use for many classical psychological experiments, including tasks using timing measurements (e.g., Crump et al., 2013; Enochson and Culbertson, 2015). For the specific case of acceptability ratings, a large-scale comparison between laboratory-based and AMT-based acceptability ratings conducted by Sprouse (2011) concluded that acceptability data collected in AMT are almost indistinguishable from laboratory data (see also Gibson et al., 2011).

### Materials

The materials used in this task were the same 36 sets of experimental items and 72-filler sentences that were used in Experiment 1. The ratio of grammatical-to-ungrammatical sentences was balanced so that half of the sentences across the task contained ungrammaticalities. During the delivery of the instructions, participants were asked to complete six practice items to ensure that they had understood the procedure.

### Procedure

The stimuli were delivered using Ibex (Drummond, 2013). Participants were presented with the entire sentence in the middle of the screen along with a rating scale. Each sentence was presented in an individual screen and participants could only move to the next one once they had emitted a rating by clicking on the scale numbers or, alternatively, using the numbers on their keyboard. Participants were instructed to rate the sentences according to their acceptability in a 7-point scale in terms of whether they came across as well-formed English: 7 meaning totally acceptable and 1 totally unacceptable. In order to help them adjust to the scale, the first two practice items were followed by feedback on “the rating that most people would give in that case” (1 or 2 for an ungrammatical example and 6 or 7 for a grammatical one). They were encouraged to take as much time as they needed and to use the entire range of the scale. The order of presentation of experimental items and fillers was randomized for each participant. The task was completed by all participants in less than 30 min.

### Analysis

The ratings collected were analyzed using a linear mixed-effects model that included the experimental conditions as fixed effects and participants and items crossed as random effects. A maximal model with a fully specified random effects structure was initially built. This model failed to converge and the random structure was simplified following Barr et al. (2013). The results reported in the next section correspond to the model with the maximal converging random effects structure, which included by-subject and by-item random intercepts and slopes but no correlation parameters for the by-item grouping. Using a log-likelihood ratio test, this model was compared to a simpler model containing only random intercepts. The test revealed that the maximally converging model provided a better fit to the data (*X*^2^_(11)_ = 72.37, *p* < 0.0001). An effect was considered to be statistically significant at the level of *p* < 0.05 when the absolute *t* value was above 2 (Gelman and Hill, 2006; Baayen et al., 2008).

### Results

**Figure 3:**
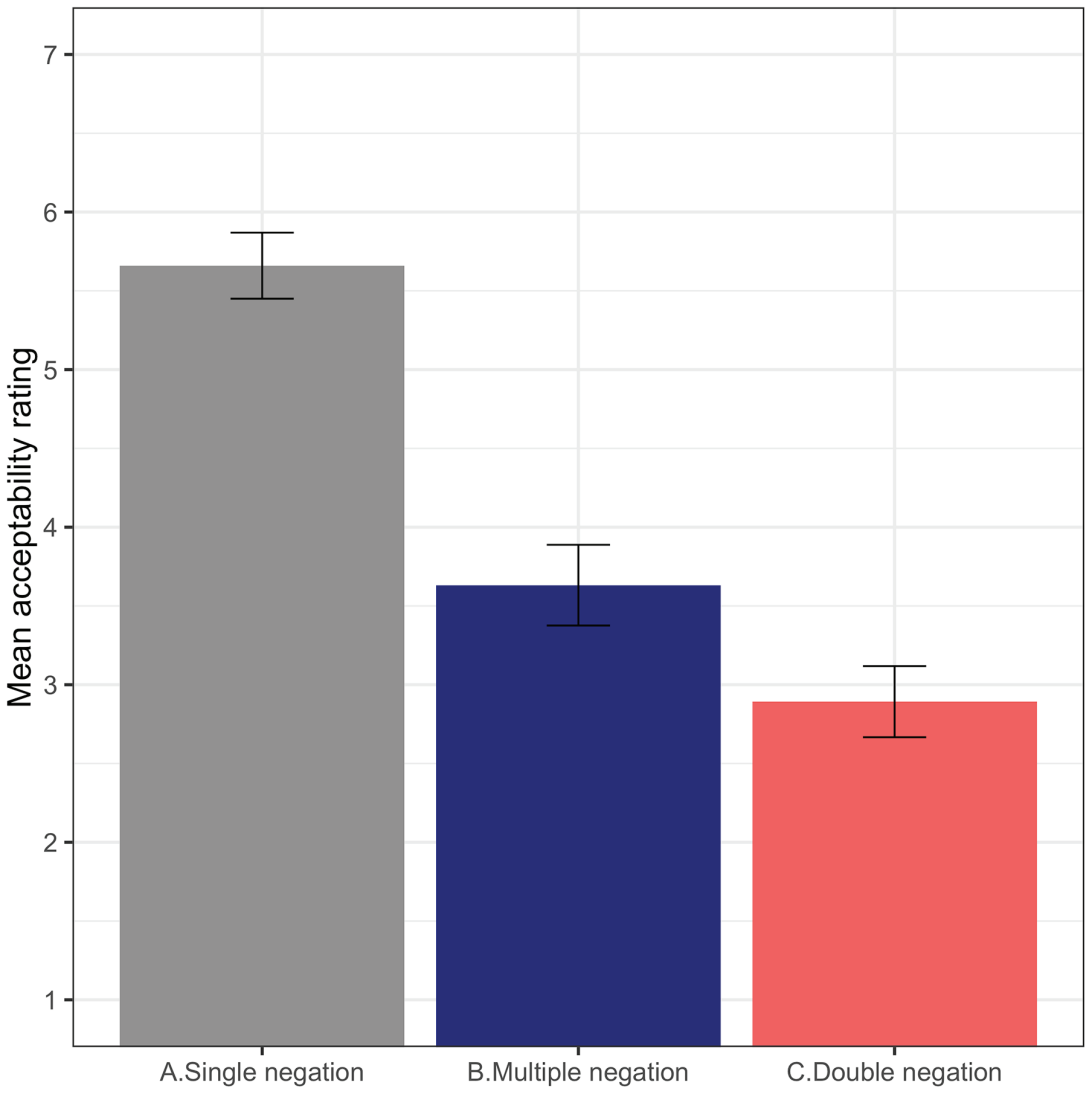
Average acceptability rating for the experimental conditions aggregated by participant (Experiment 3). Error bars indicate standard error of the mean.

The results from this experiment are presented in Figure 3. Single negation sentences had the highest average rating and double negation sentences the lowest. The acceptability of multiple negation sentences was rated quite low (means: A = 5.66, B = 3.63, C = 2.89). The model results revealed statistically significant differences among the three experimental conditions (A vs. B: $\overset{\wedge }{\beta }$ = −2.02, SE = 0.18, *t* = −11.15; A vs. C: $\overset{\wedge }{\beta }$ = −2.76, SE = 0.28, *t* = −9.93; B vs. C: $\overset{\wedge }{\beta }$ = −0.74, SE = 0.21, *t* = −3.46).

### Discussion

The first thing to note about these results is that they confirm the grammatical status that was initially assumed for the baseline conditions: while single negation sentences were judged as perfectly grammatical, double negation sentences were highly rejected. This result is unsurprising in light of Experiments 1 and 2, and it also coincides with the low acceptability ratings reported for double negation sentences in Blanchette (2015). Nonetheless, Experiment 3 was mainly designed to test whether native speakers of English recognize multiple negation sentences as acceptable in spite of the attested processing problems they generate. If these grammatical sentences were recognized as such, the ratings attributed to them should approach those of single negation sentences. However, the results from this task confirm the opposite: the perception of multiple negation sentences is highly degraded compared to single negation sentences. Multiple negation sentences patterned closer to double negation ones, although mean ratings were still lower for the latter. The key finding from this experiment is that native speakers of English fail to recognize multiple negation sentences as acceptable even though they are perfectly grammatical. This finding is relevant in several ways.

First, under the *changing encodings hypothesis,* participants are expected to access a fully encoded final-stage interpretation when given ample time. Therefore – in parallel with the pattern observed for NPI illusions – multiple negation sentences were expected to be recognized as acceptable in offline acceptability tasks as final-stage computations are supposed to be available. Instead, there is a clear conflict between grammatical knowledge and offline judgments. The results show an interesting alignment between online and offline responses, and this may question the interpretation of the findings as an illusion of ungrammaticality; at least considering a narrow definition of grammatical illusions. In the general discussion, we will put together the results from the three experiments and examine what they tell us about parser-grammar misalignments and grammatical illusions. Nonetheless, it is important to note that processing principles alone may not be enough to account for the penalized ratings of multiple negation sentences. Extralinguistic factors related to the stigmatization of negative concord varieties of English and prescriptive bans against the use of double negation (Nevalainen, 2006; Horn, 2010) could have contributed to the surprisingly low ratings attributed to these grammatical sentences; particularly in an experimental design in which they are intermixed with actual double negation sentences. In this context, the mere presence of two negative elements could have guided participants’ decisions even when they had unlimited time to provide a response.

Second, the fact that multiple negation sentences are highly penalized in offline ratings provides the strongest case against the *ever*-*never confusability hypothesis*. The processing effects observed in Experiments 1 and 2 provided initial support against this account. Yet, the different baselines used in this research (single and double negation) and in classic NPI illusions (licensed and unlicensed NPIs) make it difficult to straightforwardly map the online behavior of multiple negation sentences to NPI illusion sentences. Likert scales, the dependent variable in offline ratings, provide a slightly less baseline-conditioned indication of the perceived status of multiple negation sentences. Even though single and double negation sentences act as anchors, the experimental conditions were also intermixed with other grammatical violations that helped participants setting a threshold. Still, multiple negation sentences were given a mean rating of 3.6/7, a score that is on a par with the mean obtained by ungrammatical fillers. These results provide robust evidence that sentences containing *never* instead of *ever* are highly dispreferred by native speakers of English. The fact that they are not be able to recognize them as grammatical is in conflict with the idea that such representations could somehow rescue NPI illusions in processing tasks.

## General Discussion

The series of experiments presented here used online (i.e., speeded judgments and self-paced reading) and offline (i.e., acceptability judgments) methods as a means to study different grammatical configurations of negative elements. The focus of the project was on multiple negation sentences – condition B, repeated in (11) – which displayed the negative markers *no* and *never* in different clauses. The primary objective of this project was studying the online and offline perception of these sentences. To this end, we compared them with similar sentences without the negative element in the relative clause (i.e., single negation, condition A), and with sentences in which both *no* and *never* appeared in the main clause (i.e., double negation, condition C). The observed pattern of results was consistent across experimental measures in showing that multiple negation sentences incurred in an increased processing cost (Experiments 1 and 2) and were also perceived as less acceptable (Experiments 1 and 3) than equivalent single negation sentences. Importantly, the responses for the double negation condition across the three tasks indicate a more degraded perception and slower recovery from disruption.

(11) The authors [that **no** critics recommended] have **never** received an acknowledgement for a best-selling novel.

The fact that double negation sentences were strongly rejected confirms the initial assumptions to conceive them as a degraded baseline. Moreover, including this manipulation in the design was interesting in itself, given the limited attention that the phenomenon of double negation has received in psycholinguistics. Apart from Schiller et al. (2017), who focused on simpler combinations of verbal and affixal negation (e.g., *not unhappy*), this is, to the best of our knowledge, the first psycholinguistic study that uses time-sensitive measures to investigate double negative dependencies. Even though Standard English is commonly classified as a double negation language, this research shows that double negative dependencies do not come at free cost for the language user. This is not surprising considering that the pragmatic function of double negation is to contradict or correct a previous negative statement (Horn, 1991; Puskás, 2012), and thus, double negatives are subject to restricted pragmatic licensing conditions. As described in the introduction, double negatives have been found to appear in specific information structure configurations (Larrivée, 2016) and to be signaled by certain prosodic cues such as contradictory contour (Espinal and Prieto, 2011; Prieto et al., 2013). In addition, this investigation provides evidence that native speakers display strong processing disruptions when double negation dependencies are encountered in isolation. This finding emphasizes the mentioned pragmatic licensing requirements as a condition for double negatives to be interpreted, placing the grammar of double negation at the interface of syntax and pragmatics.

The result that participants consistently reject double negative dependencies overrules one potential concern of this research: the possibility that the participants in the experiments had grammars that allowed negative concord configurations. Native speakers of English are often exposed to instances of negative concord dependencies (e.g., *I cannot get no satisfaction*) as they are allowed in many contemporary varieties of English (e.g., African American Language or Appalachian English). In fact, some theoretical proposals (e.g., Zeijlstra, 2004; Tubau, 2008; Blanchette, 2013, 2015) have hypothesized that the underlying structure of Standard English is that of negative concord. In this vein, Blanchette and Lukyanenko (2019) demonstrate that, in the absence of the necessary licensing conditions, native speakers of English can actually interpret double negation dependencies as negative concord. The participants in our experiments were not explicitly tested for having grammars that allowed negative concord dependencies in order to avoid calling attention to the manipulation. However, we assume that interpreting double negation conditions as a case of negative concord should have facilitated its processing. On the contrary, the results regarding multiple negation and double negation conditions are the opposite to what one would expect if participants’ grammars allowed for negative concord structures. Nonetheless, the strong reactions against double negation were possibly exacerbated by two factors: first, *no* and *never* are not a frequent negative concord or double negation configuration. Second, the participants in the tasks were university educated speakers of English. As Thornton et al. (2016) pointed out, people in academic settings are generally aware of the social stigma associated with negative concord and with prescriptive views on double negation. In sum, the empirical evidence does not support the possibility that participants could be parsing the two negative elements as forming a negative concord dependency.

The main aim of this research was to test two contrasting predictions made for multiple negation sentences on the basis of previous NPI illusion accounts. On the one hand, the *ever-never confusability hypothesis* predicted that these sentences should come across as well-formed in English, and accordingly, they should be processed without problems. The results from the three experiments provide compelling evidence against this hypothesis. On the other hand, based on the *changing encodings hypothesis,* it was predicted that the negative quantifier inside the relative clause could interfere with the integration of *never*, generating an illusion of ungrammaticality. Under this rationale, despite the online interference, it was initially assumed that comprehenders should recognize multiple negation sentences as acceptable when given ample time. Instead, multiple negation sentences are consistently given low ratings in the untimed judgment task, making it less straightforward to map the relation between multiple negation sentences and NPI illusions. The connection between the two phenomena and the possible sources of the degraded perception of grammaticality is explored below.

### Relating Multiple Negation Sentences to Negative Polarity Item Illusions

Parker and Phillips’ (2016) account of NPI illusions explained spurious licensing as the consequence of accessing incomplete representations of the relative clause material when the NPI is encountered. Their account shifted the attention from the previously proposed erroneous application of NPI-specific licensing mechanisms (i.e., Vasishth et al., 2008; Xiang et al., 2009, 2013) to changes in the encoding of the representations that are used for licensing. In doing so, they provided the basis for an interesting parallelism between NPI illusion sentences and similar sentences containing *never*: if the negative quantifier is accessible to spuriously license the NPI when *ever* is encountered soon after the relative clause, it may also be accessible when *never* is encountered in the same position. The slow RTs observed for multiple negation sentences at the pre-critical region are taken as evidence that at least some aspects of the relative clause material are still being encoded, and thus, that individual feature values – such as negation – could still be transparently accessible. Even though the adverb *never*, as sentential negation, does not need to be licensed by a dependency with any previous element, under a cue-based architecture it assumed that “each incoming words triggers retrievals to integrate that word with the preceding structure” (Lewis et al., 2006, p. 448). If the embedded negation is active when *never* is being integrated, we speculate that the observed difficulties could be indexing the parser’s evaluation of a possible dependency between *no* and *never*. Given that double negative dependencies are shown to generate strong processing problems, similar problems are expected to emerge if the parser entertains a relation between *no* and *never* in multiple negation sentences.

The disruptions observed in Experiments 1 and 2 are compatible with this interpretation, and we argue that they could be understood as an illusion of ungrammaticality. Nonetheless, if this phenomenon represents the opposite case of NPI illusions, it may be initially surprising that comprehenders are unable to perceive multiple negation sentences as acceptable in untimed ratings, since they are uncontroversially grammatical. How are the low ratings explained, then? Even though offline judgments are generally conceived as a measure of acceptability, it is widely known that they are sensitive to issues of processability and have been reliably used to uncover processing effects (e.g., Fanselow and Frisch, 2006; Sprouse, 2008; Hofmeister et al., 2013; Dillon et al., 2017). With this in mind, the low ratings for multiple negation sentences could arise from the difficulties integrating *never* in the context of *no*, particularly if a temporary double negative dependency is being temporarily entertained, prompting participants to give low ratings based on simpler heuristics such as the mere presence of two negative elements. In this way, the results from Experiments 1 to 3 are compatible with an interpretation in terms of illusion of ungrammaticality. Yet, there is an alternative – and perhaps simpler – account that deserves exploring: the disruption observed for multiple negation sentences could simply reflect the parser’s limitations in processing sentences with two negations.

Integrating a negation is a complex operation that is known to impact the incremental interpretation of sentences. In multiple negation sentences, the parser must undergo this process twice: first inside the relative clause and, then, in the main clause. Processing difficulty, understood as a measure of the resources required to compute the correspondences between forms and meanings (Culicover, 2013), can accumulate during sentence processing in such a way that it can produce additive effects (e.g., Gibson, 1990; Kluender and Kutas, 1993). Thus, one could speculate that the comprehension system may not be able to handle the additive syntactic, semantic and pragmatic complexity of two negative operations when they appear close in the input. In multiple negation sentences, this processing overload is expected to originate when the second negation (i.e., *never*) is encountered if the first negation (i.e., *no*) is still being integrated, exceeding the computational resources of the system. As a consequence, grammar-independent factors related to the limitations of human parser may impede the identification of the correct grammatical analysis, resulting in the processing problems and low acceptability ratings observed. Assuming that processing complexity alone can account for the results eliminates the need to appeal to intermediate stages of representation building and the temporary evaluation of a dependency between *no* and *never* as the source of the effects. In some respects, this interpretation of the findings treats multiple negation sentences on a par with other patterns of misalignment like multiple center embeddings^8^. Indeed, some authors (e.g., Bever, 1970; Corblin, 1996) have conceptually associated the complexity of negation to that of multiple embedding. Multiple center-embedding sentences reflect the limitations of the parser to generate a representation that is nonetheless available in the grammatical repertoire. Along the same lines, multiple negation sentences could represent another instantiation of the computational limitations of the comprehension system.

If an explanation based solely on processing complexity is the right characterization of the empirical evidence, this limitation of the human parser is expected to extend to similar sentences containing two negative markers. Nonetheless, a number of observations suggest that native speakers of English are able to generate valid representations for sentences that contain two negative elements. For instance, speakers of English can, presumably, understand and express sentences like (12) in spite of their relative complexity.

(12) I did not promise that I would not go.

Sentences like (12) are unsurprising from the perspective of theoretical linguistics because each negative element can only be interpreted independently and, thus, each clause illustrates an instance of single negation. This may explain why these type of constructions are only mentioned in passing by theoretical linguistic works, which describe them as unproblematic and frequent in natural languages (Huddleston and Pullum, 2002; Zeijlstra, 2004). In a recent work using the truth-value judgment task (Crain and Thornton, 1998), Thornton et al. (2016) compared adult and children’s interpretation of sentences with double negation and negative concord dependencies. In order to assess the possibility that children could experience problems with two negations simply due to processing limitations, they included sentences like (13) as a control condition.

(13) The girl who did not skip bought nothing.

Similar to our multiple negation sentences, the control condition in Thornton et al. (2016) contained two independent negative markers in different clauses: one inside a relative clause (i.e., *did not*) and the other in the main clause (i.e., *nothing*). If an explanation based on processing is on the right track these sentences are expected to be problematic. However, the results by Thornton et al. (2016) do not seem to point in this direction, as neither adults nor children exhibited problems with them. Importantly, though, the task in Thornton et al. (2016) was a truth-value judgment, which was presented in a context. Although further research should consider this more carefully, the evidence so far suggests that native speakers of English can indeed parse sentences with two negative markers, and thus, that multiple negation sentences and multiple center-embedding should not be conceptualized as analogous cases. Furthermore, there are two remarkable differences between Thornton et al.’s controls and our multiple negation sentences that strengthen the parallelisms with NPI illusions. First, in (13), the main clause negation *nothing* appears after the main clause verb (i.e., *bought*). In our stimuli, *never* appears before the verb, and thus, closer to the relative clause. This is an interesting fact if we take into account that Parker and Phillips’ (2016) study demonstrates that illusory licensing disappears when the unlicensed NPI is located after the main clause verb (see example 9). Second, whereas the intervening negation in multiple negation sentences is a negative quantifier, the control sentences by Thornton and colleagues use verbal negation. In a recent investigation, de-Dios-Flores et al. (2017) found that the classic NPI illusion pattern does not occur when the intervening negation is verbal negation, suggesting that NPI illusions arise at least in part as a result of the use of quantificational licensors in the relative clause (cf. Muller et al., 2019).

Considering the above, it is possible that differences in the type and relative position of the negations could explain the contrast between the difficulties generated by multiple negation sentences and the apparent ease with which sentences like (13) are interpreted by both adults and children. These observations about NPI illusions generate interesting predictions for multiple negation sentences. In particular, further research should clarify the role of distance and type of negation in the processing problems observed in multiple negation sentences and also the possible interpretations that speakers ascribe to multiple and double negation sentences. In light of the above, it seems unlikely that the processing problems and degraded perception of multiple negation sentences are solely explained by the additive complexity of integrating two negations. Indeed, if comprehenders were unable to deal with these sentences simply because they have two negations, multiple and double negation sentences should pattern alike in the three tasks. Contrary to this, the differences between these two conditions is patent across tasks and measurements. This is particularly evident in Experiment 1, in which multiple negation sentences were accepted in more than 60% of the cases whereas the acceptance of double negation sentences was below 30%.

The degree of similarity between NPI illusions and multiple negation suggests that the same incomplete encodings that ameliorate the online perception of unlicensed NPIs could be responsible for deteriorating the online perception of grammatical multiple negation sentences. This interpretation of the results generalizes Parker and Phillips’ (2016) account of NPI illusions to other configurations, with the additional assumption that the low ratings are the combined product of these processing difficulties and simpler heuristics such as the mere presence of two negative elements. Such heuristics could have been developed by participants as a consequence of the existing social stigmas and prescriptive bans against negative concord and double negation. The hypothesized intrusion of extralinguistic pressures is supported by the fact that multiple negation sentences were actually more penalized when participants had unlimited time (Experiment 3) than when they were asked to provide fast judgments (Experiment 1). By way of conclusion, the next section tries to integrate these findings in the broader context of misalignments.

### Widening the Grammatical Illusions Landscape

This research has taken NPI illusions as a starting point in order to examine a candidate structure for a case of illusion of ungrammaticality. To this end, our stimuli were created by substituting the NPI *ever* in Parker and Phillips’ (2016) illusion stimuli by the negative adverb *never*. The results confirm that the integration of the adverb *never* in the main clause is disrupted by the presence of a linearly preceding but structurally inaccessible negative quantifier, resulting in perceived unacceptability of grammatical sentences. The previous section discussed two possible explanations for this interesting pattern of misalignment: one possibility is that they reflect an arbitrary failure of the system due to processing complexity. Another possibility is that the problems attested in multiple negation sentences can be predicted from the same erroneous computations that cause NPI illusions. Our evaluation of the evidence points to the latter, although further research is necessary in order to clarify the degree of similarity between the two phenomena. Either way, multiple negation sentences represent a hitherto unknown case of misalignment that opposes grammatical knowledge with online/offline responses. Conceptualizing it as an illusion of ungrammaticality invites a reflection on the definition and scope of the concept of grammatical illusions.

In the introductory section of this paper, agreement attraction and the spurious licensing of NPIs were presented as paradigmatic examples of grammatical illusions. In this context, the concept of grammatical illusions is generally reserved to describe cases in which grammatical violations do not seem to be perceived in online measures but are then perfectly identified when comprehenders are given ample time. This characterization of grammatical illusions comes with two important assumptions: first, that illusory processes do not affect offline ratings, and second, that comprehenders are not thought to experience the opposite phenomenon (i.e., illusions of ungrammaticality). Even though agreement and NPI illusions often fit into this narrow definition, careful examination of the empirical evidence does not always support such a neat characterization. With regard to the first assumption, previous studies have actually reported an improved perception for NPI illusion sentences also in acceptability judgments, even when the amelioration effects are much weaker than those obtained in online tasks (e.g., Xiang et al., 2006; de-Dios-Flores et al., 2017; Yanilmaz and Drury, 2018)^9^. Thus, there is evidence that grammatical illusions do sometimes affect offline ratings. In addition, comparative illusions (Wellwood et al., 2018) and the presence of agreement attraction effects in production tasks (Bock and Miller, 1991; Bock et al., 2012) are other examples that grammatical illusions do not always surface as neat differences between online and offline responses. With regard to the second assumption, in addition to the evidence collected in this project, there are examples in the literature that could be classified as illusions of ungrammaticality. For instance, in the study of agreement dependencies, some researchers have reported that attraction effects also affect agreement relations in grammatical sentences (e.g., Acuña-Fariña et al., 2014; Lago et al., 2015; Laurinavichyute and von-der-Malsburg, 2019). Along the same lines, it has been shown that the perception of perfectly grammatical unagreement dependencies is degraded in online measures (Mancini et al., 2014; Mancini, 2018).

This varied pattern of misalignments challenges the narrow definition of grammatical illusions because it leaves out many interesting effects, limiting the characterization of the existing evidence and our understanding of the connections among different phenomena – e.g., between multiple negation sentences and NPI illusions. If linguistic illusions are understood as mismatches between grammatical knowledge and the outcomes of language comprehension, a wider illusory space should include misalignments that affect both grammatical and ungrammatical sentences as well as permanent (i.e., online and offline) and temporary (i.e., online) effects. Such a broader conceptualization of illusion-like phenomena would not need to capitalize on black and white distinctions between online and offline responses, while it should still delve deeper on the reasons why different types of dependencies yield different patterns of misalignment in online and offline tasks. Specific linguistic configurations – like multiple negation sentences or NPI illusions – are not ultimately investigated in order to understand them in isolation; but rather, to understand their connections and integrate them into a theory of how misalignments emerge.

## Data Availability Statement

The datasets generated for this study are available on request to the corresponding author.

## Ethics Statement

Ethical review and approval were not required for the study on human participants in accordance with the local legislation and institutional requirements. The patients/participants provided their written informed consent to participate in this study.

## Author Contributions

The author confirms being the sole contributor of this work and has approved it for publication.

### Conflict of Interest

The author declares that the research was conducted in the absence of any commercial or financial relationships that could be construed as a potential conflict of interest.

## Footnotes

^1^Questions, conditionals, or comparative structures are also frequent licensing environments (see Giannakidou, 2011, for a recent review).

^2^Given that speeded acceptability tasks present a two-alternative forced choice (yes/no), the three-way distinction arises because not all the participants experience an allusion for all the items.

^3^Note that the post-verbal placement of *any* is enough distance in order to turn the illusion off.

^4^As noted by an anonymous reviewer, under a noisy-channel architecture, it is possible that *never* is misrepresented as *ever* in cases in which the later allows a more probable structure (e.g., *No one never came*). Nonetheless, as an explanation of NPI illusions, the *ever-never confusability hypothesis* is not assumed to go both the ways. If substituting *ever* with *never* is what is thought to improve the perception of the unlicensed NPI *ever* in illusion sentences, then *never* is assumed to be parsed without problems.

^5^The materials included combinations of sentential negation with affixal and semantic negation (e.g., *not, no one, doubted, un-*).

^6^https://www.edu.xunta.gal/portal/es/linguasestranxeiras/1640/1643

^7^It is relevant for the discussion to report that when trial order is included in the model, the contrast between conditions A (single negation) and B (multiple negation) in the pre-critical region emerged statistically significant. This contrast had not reached statistical significance in the model results reported for Experiment 2.

^8^Example (4) repeated here: *The patient who the nurse who the clinic had hired met Jack.

^9^It is important to note that not every study on NPI illusions reports acceptability ratings for their stimuli (e.g. Vasishth et al., 2008; Xiang et al., 2009).
